# Approaching the taxonomic affiliation of unidentified sequences in public databases – an example from the mycorrhizal fungi

**DOI:** 10.1186/1471-2105-6-178

**Published:** 2005-07-18

**Authors:** R Henrik Nilsson, Erik Kristiansson, Martin Ryberg, Karl-Henrik Larsson

**Affiliations:** 1 Göteborg University, Botanical Institute, Box 461, 405 30 Göteborg, Sweden; 2 Chalmers University of Technology, Mathematical Sciences, 412 96 Göteborg, Sweden

## Abstract

**Background:**

During the last few years, DNA sequence analysis has become one of the primary means of taxonomic identification of species, particularly so for species that are minute or otherwise lack distinct, readily obtainable morphological characters. Although the number of sequences available for comparison in public databases such as GenBank increases exponentially, only a minuscule fraction of all organisms have been sequenced, leaving taxon sampling a momentous problem for sequence-based taxonomic identification. When querying GenBank with a set of unidentified sequences, a considerable proportion typically lack fully identified matches, forming an ever-mounting pile of sequences that the researcher will have to monitor manually in the hope that new, clarifying sequences have been submitted by other researchers. To alleviate these concerns, a project to automatically monitor select unidentified sequences in GenBank for taxonomic progress through repeated local BLAST searches was initiated. Mycorrhizal fungi – a field where species identification often is prohibitively complex – and the much used *ITS *locus were chosen as test bed.

**Results:**

A Perl script package called *emerencia *is presented. On a regular basis, it downloads select sequences from GenBank, separates the identified sequences from those insufficiently identified, and performs BLAST searches between these two datasets, storing all results in an SQL database. On the accompanying web-service , users can monitor the taxonomic progress of insufficiently identified sequences over time, either through active searches or by signing up for e-mail notification upon disclosure of better matches. Other search categories, such as listing all insufficiently identified sequences (and their present best fully identified matches) publication-wise, are also available.

**Discussion:**

The ever-increasing use of DNA sequences for identification purposes largely falls back on the assumption that public sequence databases contain a thorough sampling of taxonomically well-annotated sequences. Taxonomy, held by some to be an old-fashioned trade, has accordingly never been more important. *emerencia *does not automate the taxonomic process, but it does allow researchers to focus their efforts elsewhere than countless manual BLAST runs and arduous sieving of BLAST hit lists. The *emerencia *system is available on an open source basis for local installation with any organism and gene group as targets.

## Background

**Figure 1:**
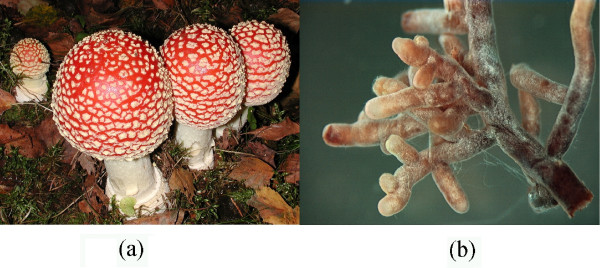
**The fly agaric: a common mycorrhizal fungus. **a) Fruiting-bodies of the ectomycorrhizal fly agaric *(Amanita muscaria)*. b) Root-tip mycelia of the *Amanita *type.

Mycorrhiza is the term used to denote the root-associated symbiosis between fungus and plant where resources otherwise unattainable or very costly (chiefly carbohydrates and mineral nutrients, respectively) are exchanged. Whereas the identification of the plant partner of the symbiosis often is comparatively straightforward, the identity of the fungal component is typically much more elusive, largely so due to the dearth of information obtainable from the root samples of the plant (Figure 1). The traditional approaches to identification of mycorrhizae include studies based on light microscopy, isozyme assays, mating behaviour experiments, and somatic compatibility tests. However, all of these are associated with drawbacks such as low to moderate precision, high time consumption, or the requirement that the fungus be isolated and grown in culture, which is impossible for many mycorrhizal fungi [1-4]. Alternatively, it is sometimes possible to establish a hyphal connection between the fungal mycelium of the root tips and nearby fungal fruiting-bodies [5]; traditional fungal taxonomy rests to a large extent on the morphology of fruiting-bodies or other spore producing structures, and there is abundant literature information available for many groups of fungi. The factors triggering the formation of fruiting-bodies of mycorrhizal fungi are, however, poorly understood. This is reflected in the large number of root-associated fungi for which fruiting-bodies have never been found, suggesting that any attempt to characterize the below-ground mycoflora through collection and identification of above-ground fruiting-bodies is likely to give a skewed and incomplete picture [6-8].

The advent of PCR-based methodologies has in many ways revolutionized the field of mycorrhizal research by providing a means by which, at least in theory, every fungus could be identified to species level. It has also served to highlight further the patchiness in our understanding of mycorrhizal fungi; DNA-based studies of mycorrhizal fungi typically contain a considerable proportion of sequences that lack identified matches in public sequence repositories such as GenBank and UNITE [9], and that hence cannot be identified to species or even genus level [10-13]. The prerequisite of most journals that all sequences used in a publication be made public in GenBank naturally leads authors to submit such unidentified sequences under names like "Fungus: Environmental sample", "Unidentified mycorrhizal basidiomycete", "Uncultured root-tip fungus", and *"Cortinarius *sp.". Thus, even if an unidentified mycorrhizal sequence turns out to have one or more identical (or nearly so) matches in GenBank, the unidentified nature of the latter still precludes identification to species level.

As more and more sequences from well-identified fruiting-bodies are submitted to and accumulate in GenBank, one can expect some of the new sequences to hint and even resolve the identity of previously unmatched sequences. Any such relation is likely to impart important information to the studies that employed the unidentified sequences in question. Yet there is no generic mechanism on GenBank to alert the sequences' authors – or anyone else who might be interested – of these new matches. In addition, many sequence authors are inefficient in keeping the annotations of their submitted sequences up-to-date, even when new taxonomic progress and knowledge have been attained. As a result, important information goes by undetected, and obsolete annotations remain and are propagated through the databases and literature via subsequent BLAST runs [14]. The present study introduces *emerencia*, a Perl script package to facilitate the keeping track of the identity of insufficiently identified GenBank sequences over time. A web-service demonstrating *emerencia * for mycorrhizal fungi and the nuclear *ITS *region is presented; *emerencia *is also available on an open source-basis and can be downloaded from the web page for local installation with any gene and organism group as targets.

## Implementation

The web-service provided at  is set to monitor the identity of fungal *ITS *sequences whose taxonomic annotations are poorly resolved. The *ITS *region offers three sub-loci of very different conservation levels *(ITS1- *very variable; *5.8S- *very conserved; *ITS2- *semi-conserved [15]) and is as such the prevalent region of choice when fungi are to be identified [16,17,3].

The main script of the *emerencia *package is written in Perl [18] and runs in a UNIX environment. On a regular basis, the script is evoked and connects to GenBank through BioPerl routines [19] to retrieve all fungal *ITS *sequences deposited since the last update using the query phrase

(("Fungi" [Organism] AND (200 [SLEN] : 3000 [SLEN])) AND (((ITS1 [titl] OR ITS2 [titl]) OR 5.8S [titl]) OR "internal transcribed spacer" [titl] OR "internal transcribed spacers" [titl]))

For each downloaded sequence, the GenBank format annotation is parsed to determine whether the sequence should be regarded as fully identified (i.e., identified to species level) or insufficiently identified (i.e., identified only to phylum Fungi (e.g., "Unidentified fungus"), identified to ordinal level (e.g., *"Thelephorales *sp."), or identified to generic level (e.g., *"Amanita *sp.")). The criteria for this decision can be found in full detail at ; for example, a sequence is regarded as insufficiently identified if its species annotation (GenBank format reference: SOURCE) contains words like "unidentified", "uncultured", "sp.", "mycorrhizal isolate", or "unknown" (this section of the script was repeatedly fine-tuned to minimize the number of false positives and negatives). All new sequences are appended to one of two tables of a local MySQL database [20] such that one table holds the identified sequences and the other the insufficiently identified ones. The structure of the database is provided at . Local BLAST search indices [21] are computed to allow for similarity searches in both tables.

Two sets of local BLAST searches are then run using default settings:

**1. **all insufficiently identified sequences are BLAST:ed against all identified sequences

**2. **all insufficiently identified sequences are BLAST:ed against all insufficiently identified sequences.

For the first BLAST run (**1**), details of the best BLAST match are inspected. If, for any insufficiently identified sequence, the best BLAST match to the table of identified sequences is found to have changed (i.e., a change in the accession number of the best BLAST match in combination with an improved E-value or identical E-value and an improved score) – or if the unidentified sequence lacks any previous significant match – details of the new best BLAST match (accession number, date, and BLAST score and E-value) are appended to the entry of the insufficiently identified sequence. Similarly, for the second BLAST run (**2**), the best non-self match of each insufficiently identified sequence to the table of insufficiently identified sequences is noted and saved. To retain a BLAST history, the former best BLAST match of each insufficiently identified sequence is also kept. The end product of the main script of *emerencia *is, thus, two updated, mutually exclusive MySQL tables – one with identified fungal *ITS *sequences and one with insufficiently identified *ITS *sequences, and both with cross-linked indices on best and former best BLAST matches in each table.

The web-service enables visitors to interact with the database in a number of ways. Four major search categories are provided (Table 1):

**Table 1:** Functions of the *emerencia *web-service. Functions of the *emerencia *web-service at ; some examples and an informal walkthrough are also given at this address. The output of the functions features relayed hyperlinks to GenBank, Google, and Tree of Life for quick retrieval of additional information; where applicable, insufficiently identified sequences are also hyperlinked to the SEARCH FOR INSUFFICIENTLY IDENTIFIED SEQUENCE BY ACCESSION NUMBER function for a more detailed description of the sequence and its matches.

SEARCH FOR INSUFFICIENTLY IDENTIFIED SEQUENCE BY ACCESSION NUMBER	For any given accession number of an insufficiently identified sequence, this function shows the present and previous best BLAST matches from the table of identified sequences together with match scores and relevant annotation. A Clustal W multiple alignment [34] of the sequences is generated and shown as an important aid in interpreting the BLAST match values. In addition, all the above is shown for the present and previous best BLAST matches in the table of insufficiently identified sequences. This function requires that the accession number provided by the user be present in the table of insufficiently identified sequences.
CHECK SPECIFIC PUBLICATION FOR INSUFFICIENTLY IDENTIFIED SEQUENCES AND THEIR IDENTITY	This function retrieves all insufficiently identified sequences stemming from the user-specified publication and shows the present best identified BLAST match (and some additional information) for those sequences. The function expects 5–10 distinct key words from the title / author /journal fields of the publication and requires that at least one insufficiently identified sequence was released together with the publication in question.
SEARCH FOR INSUFFICIENTLY IDENTIFIED SEQUENCES MATCHING ACCESSION NUMBER OF IDENTIFIED TAXA	For any given accession number in the table of identified sequences, this function retrieves and details all entries in the table of insufficiently identified sequences for which this accession number represents the best BLAST match. It requires that the specified accession number be present in the table of identified sequences and will proceed only if that accession number indeed constitutes the best BLAST match of at least one insufficiently identified sequence.
SEARCH FOR INSUFFICIENTLY IDENTIFIED SEQUENCES BY KEY WORD	This function lets the user query the species annotation field of the table of insufficiently identified sequences using 2–5 key words, and displays all insufficiently identified sequences matching the key words. For those sequences, the best BLAST match to the table of identified sequences will be shown together with some additional information.

• SEARCH FOR INSUFFICIENTLY IDENTIFIED SEQUENCE BY ACCESSION NUMBER

• CHECK SPECIFIC PUBLICATION FOR INSUFFICIENTLY IDENTIFIED SEQUENCES AND THEIR IDENTITY

• SEARCH FOR INSUFFICIENTLY IDENTIFIED SEQUENCES MATCHING ACCESSION NUMBER OF IDENTIFIED TAXA

• SEARCH FOR INSUFFICIENTLY IDENTIFIED SEQUENCES BY KEY WORD

The different search functions produce output pages that are extensively hyperlinked to facilitate further queries against the web-service itself as well as external information resources such as GenBank and the Tree of Life project [22]. Apart from querying the database through a web browser and bookmarking or saving the results, users can subscribe to accession numbers of insufficiently identified sequences. This enables immediate notification by e-mail when the best BLAST matches of those sequences change.

The web-service is hosted on a MacOS X server running the Apache web server [23]. The databases are queried using dedicated CGI scripts written in Perl; parts of the source code of the galaxieEST and *mor *packages [24,17] were used for this purpose. The extensively annotated source code of the *emerencia *core is freely available at the web-service. Local installation and additional technical aspects are described in the documentation.

## Results and Discussion

The last decade has seen a dramatic improvement of our understanding of mycorrhizal diversity, largely due to the advent of fast and comparatively cheap PCR-based methods. The discovery of new, previously unsequenced mycorrhizal fungi poses something of a taxonomic problem, particularly when fruiting-bodies and other distinguishing characteristics are absent. Many of these sequences are submitted *ad tempus *as "environmental samples". Unfortunately, the absence of generic mechanisms – and the apparent lack of motivation of the authors of the sequences – to refine the identity of these sequences as more information is amassed force other researchers to put in a great deal of effort (typically countless manual BLAST runs) in order to make sense of the sequences and the relation of their own sequences to those. With more than a handful of sequences to monitor, the task quickly becomes unreasonably time-consuming. The authors present a prototype software package to minimize the amount of work needd to stay updated on the identity of such insufficiently identified sequences. The web-service provided allows users to subscribe to accession numbers (sequences) with automatic email notification upon identity changes; alternatively, the same – and additional – information can be obtained through the search functions of the web-service. Furthermore, to install *emerencia *locally and modify it to run with other organism and gene groups should not pose any problem to anyone with a reasonable experience of UNIX-type environments. Such a local installation can stretch from a private, shell window-only tool to a public, user-oriented web-service such as the one presented here. For a local installation, parameters such as how often the script should be started, the BLAST settings, and what information to store locally, can be set as seen fit.

As of May 2005, *emerencia *has fetched about 29000 identified and 7500 insufficiently identified *ITS *sequences (Table 2). The identified sequences belong to some 8000 distinct species, which corresponds to approximately 0.5% of the estimated 1.5 million extant species of fungi [25]. While the number of fungal sequences in GenBank is expected to increase drastically over the next few years, it will take a long time before all gaps are filled [26], leaving taxon sampling a tangible problem for *emerencia *as well as for other tools used for sequence identification. In addition, the poor state of many taxonomic annotations in GenBank and other databases [27,28] complicates the above percentage estimates and poses a challenge to users of *emerencia*. As with other identification tools, it is crucial that the results obtained be viewed and used as guidance for further studies rather than accepted as true and final; *emerencia *is a tool to refine iteratively the identity of insufficiently identified sequences in public databases and to promote the flow of information pertaining to those sequences. It is not intended – and should never be used – as a shortcut to unequivocally correct species names and annotations.

**Table 2:** A brief summary of the sequence data underlying the *emerencia *web service at  as of May 2005. The threshold BLAST E-values for "good" and "poor" matches were arbitrarily set to 0.0 and 1e-100, respectively. Graphical illustrations showing the population of the database over time and additional aspects of *emerencia *are generated automatically on a monthly basis and are available at the above address.

NUMBER OF INSUFFICIENTLY IDENTIFIED SEQUENCES	7528 (21 % of total)
NUMBER OF IDENTIFIED SEQUENCES	28959 (79% of total)
NUMBER OF INSUFFICIENTLY IDENTIFIED SEQUENCES WITH GOOD MATCHES (E-VALUE = 0.0)	4791 (64 % of the insufficiently identified sequences)
NUMBER OF INSUFFICIENTLY IDENTIFIED SEQUENCES WITH POOR MATCHES (E-VALUE >1E-100)	1135 (15 % of the insufficiently identified sequences)
TOTAL NUMBER OF SEQUENCES LAST UPDATED BEFORE 1995-01-01	180 (0.5%)
TOTAL NUMBER OF SEQUENCES LAST UPDATED BEFORE 2000-01-01	3651 (10 %)
TOTAL NUMBER OF SEQUENCES LAST UPDATED BEFORE 2005-01-01	31858 (87%)
NUMBER OF INSUFFICIENTLY IDENTIFIED SEQUENCES LAST UPDATED BEFORE 2000-01-01	264 (3.5 % of the insufficiently identified sequences)
NUMBER OF INSUFFICIENTLY IDENTIFIED SEQUENCES LAST UPDATED BEFORE 2000-01-01 AND WITH POOR MATCHES (E-VALUE > 1E-100)	17 (0.2 % of the insufficiently identified sequences)
NUMBER OF IDENTIFIED SEQUENCES HAVING AT LEAST ONE INSUFFICIENTLY IDENTIFIED COUNTERPART AS IDENTIFIED BY BLAST	2981 (10 % of the identified sequences)
NUMBER OF IDENTIFIED SEQUENCES WITHOUT INSUFFICIENTLY IDENTIFIED COUNTERPARTS	25978 (90 % of the identified sequences)

As with BLAST searches in general, several factors impede the interpretation of the result. The aforementioned problem with taxonomic annotations in GenBank calls, in itself, for subsequent hands-on verification of the results. Furthermore, BLAST explores – and tries to expand – local regions of sequence similarity, and it takes manual inspection of the BLAST results to find out whether the entire, or only a portion of, the query sequence was successfully matched to anything in the database. A match to only a part of the target sequence (such as the very conserved *5.8S *sub-locus of the *ITS *region) is, for identification purposes, tantamount to no match at all [16]. It is also important to keep in mind that BLAST provides a measure of similarity, but similarity does not in turn provide a sound measure of relatedness [29,30]. Finally, it is notoriously difficult to tell an identified sequence apart from an insufficiently identified one on an automated basis; indeed, the present set-up is likely to yield a small proportion of false positives as well as false negatives (presently less than 0.1%). Such problems would largely have been avoided had there been an accepted standard for annotation of unidentified – and identified – sequences.

*emerencia *bears some resemblance to tools like Sequence Alerting System [31], Swiss-Shop [32], and ReHab [33], but a number of features set *emerencia *apart from these. *emerencia *is primarily a taxonomic utility designed to add an integrative aspect to GenBank data; its automated separation of identified and insufficiently identified sequences paves the way for researchers seeking reliable identification of species rather than merely the best possible match scores. *emerencia *can be installed locally or accessed over the Internet; in the latter case, the user will need nothing but a web browser. The data structure of *emerencia *allows many types of interesting questions to be asked; for instance, insufficiently identified sequences and their present best identified matches can be listed publication-wise, or all insufficiently identified sequences that constitute the best BLAST match of some given identified sequence can be listed (essentially amounting to a BLAST run in reverse) (Table 1). *emerencia *is tailored for variable nucleotide sequences, whereas proteins represent the target for Swiss-Shop and ReHab. Finally, the e-mail subscription utility provides a convenient way for users to stay taxonomically updated on select insufficiently identified sequences with a minimum of effort.

## Conclusion

Insufficiently identified sequences generally add little to the studies in which they are included, and it is important to estimate their identity as correctly as possible. The magnitude of this manual task increases with the number of sequences, but this process can fortunately be automated. However, in spite of computational advances, the taxonomic process itself lies beyond automation, alluding to the importance of both good species knowledge and the inherent need to always approach hypothesized identifications in a critical way.

## Availability and requirements

**Project name: ***emerencia*

**Project home page: **

**Operating system(s): **Primarily UNIX type platforms

**Programming language: **PERL, SQL

**Other requirements: **BLAST, Apache httpd, BioPerl, Clustal W (optional)

**License: **GNU GPL version 2

**Any restrictions to use by non-academics: **None other than those imposed by GNU GPL version 2

## List of abbreviations

BLAST – Basic Local Alignment Search Tool

*ITS- *Internal Transcribed Spacers

SQL – Structured Query Language

## Authors' contributions

All authors contributed to the structure and functions of *emerencia*. RHN and EK wrote most parts of the *emerencia *core, the CGI scripts, and the database handlers. MR was responsible for the mycological part, including literature comparison, integrity testing, and data verification. KHL contributed with advice on fungal taxonomy and systematics. All authors drafted the manuscript and approved the final version.
